# Phospholipase C Gamma 2 Is Critical for Development of a Murine Model of Inflammatory Arthritis by Affecting Actin Dynamics in Dendritic Cells

**DOI:** 10.1371/journal.pone.0008909

**Published:** 2010-01-27

**Authors:** Viviana Cremasco, Elisa Benasciutti, Marina Cella, Marina Kisseleva, Monica Croke, Roberta Faccio

**Affiliations:** 1 Department of Orthopaedics, Washington University School of Medicine, St. Louis, Missouri, United States of America; 2 Department of Human Anatomy and Histology, University of Bari, Bari, Italy; 3 Department of Molecular Genetics, S. Raffaele Scientific Institute, Milano, Italy; 4 Department of Pathology and Immunology, Washington University School of Medicine, St. Louis, Missouri, United States of America; 5 Department of Internal Medicine, Washington University School of Medicine, St. Louis, Missouri, United States of America; New York University, United States of America

## Abstract

**Background:**

Dendritic cells (DCs) are highly specialized cells, which capture antigen in peripheral tissues and migrate to lymph nodes, where they dynamically interact with and activate T cells. Both migration and formation of DC-T cell contacts depend on cytoskeleton plasticity. However, the molecular bases governing these events have not been completely defined.

**Methodology/Principal Findings:**

Utilizing a T cell-dependent model of arthritis, we find that PLCγ2−/− mice are protected from local inflammation and bone erosion. PLCγ2 controls actin remodeling in dendritic cells, thereby affecting their capacity to prime T cells. DCs from PLCγ2−/− mice mature normally, however they lack podosomes, typical actin structures of motile cells. Absence of PLCγ2 impacts both DC trafficking to the lymph nodes and migration towards CCL21. The interaction with T cells is also affected by PLCγ2 deficiency. Mechanistically, PLCγ2 is activated by CCL21 and modulates Rac activation. Rac1/2−/− DCs also lack podosomes and do not respond to CCL21. Finally, antigen pulsed PLCγ2−/− DCs fail to promote T cell activation and induce inflammation in vivo when injected into WT mice. Conversely, injection of WT DCs into PLCγ2−/− mice rescues the inflammatory response but not focal osteolysis, confirming the importance of PLCγ2 both in immune and bone systems.

**Conclusions/Significance:**

This study demonstrates a critical role for PLCγ2 in eliciting inflammatory responses by regulating actin dynamics in DCs and positions the PLCγ2 pathway as a common orchestrator of bone and immune cell functions during arthritis.

## Introduction

Dendritic cells (DCs) constitute a unique bridge between the innate and the adaptive immune system. They are potent antigen presenting cells with the specific ability to stimulate naïve T cells and control T cell responses [1]. For their T cell priming capacity, DCs actively participate in development of autoimmune conditions. In particular, several reports support the importance of DCs during rheumatoid arthritis, a highly inflammatory disease characterized by proliferation of synovial tissue and associated joint destruction [2]. It is generally agreed that the arthritic disease process involves abnormal presentation of self-antigens by antigen presenting cells, leading to activation of autoreactive T lymphocytes, that are a significant component of RA pathogenesis [3]. Histological studies in patients showed that DCs cluster with T cells close to blood vessels, and presence of T cell infiltrates in the synovium are often observed [4], [5], [6]. Furthermore, experimental animal studies have shown that DCs pulsed with collagen are able to induce autoimmune arthritis after transfer to joints [6], suggesting that DCs play an active role during the inductive phase of inflammation. For all these reasons, a lot of efforts have been spent in trying to elucidate the mechanisms controlling DC functions, as a way to target T cell responses.

DCs are activated in the periphery where they internalize and process the antigen [7], [8]. Next, DCs migrate to the lymph nodes where they present the antigen on MHC molecules, and the process culminates with activation of reactive T cells. DC migration is ensured by presence of podosomes, adhesive structures mediating short-lived and low-affinity interactions with the substrate. Podosomes are made by a core of actin bundles surrounded by a ring of vinculin and other actin regulatory proteins [9]. They are highly dynamic structures, fast assembled and disassembled to allow progression of the cell leading edge toward the migratory stimuli. Once DCs reach the lymph nodes, podosomes are replaced by long membrane extensions, called dendrites, which favor the interaction with potentially reactive T cells [5]. Dynamic changes in the actin cytoskeleton are controlled by the Rho family of small GTPases. In particular, Rac has been reported to be a critical player in cell motility in various cell types [10], [11], [12]. Rac1,2−/− DCs have migratory defects in vivo and fail to induce T cell priming due to impaired interactions with T cells [13]. The signaling pathway governing Rac activation in DCs is still under investigation.

Phospolipase C gamma 2 (PLCγ2), an enzyme that hydrolyzes Phosphatidylinositol(4,5)-biphosphate to generate the two second messengers inositol(1,4,5)-trisphosphate and diacylglycerol, is implicated in actin cytoskeletal reorganization in osteoclasts and neutrophils [14], [15], [16]. Importantly, PLCγ2−/− mice are protected from inflammation and consequent bone loss in the serum transfer arthritis model due to defective neutrophil activation [14]. Another consequence of PLCγ2 deficiency is decreased B cell number, while PLCγ2−/− T cells develop normally [17], [18], and defective osteoclast differentiation and function [15], [16]. Interestingly, PLCγ2 activation has also been observed in DCs in response to LPS [19], however the phenotype of PLCγ2−/− DCs, including their capacity to activate T cells, remains to be elucidated.

In this study we explored the contribution of PLCγ2 in DC functions as well as in the development of a T cell dependent model of arthritis in vivo. Our results indicate that PLCγ2 is involved in actin cytoskeleton reorganization and Rac-activation in DCs thereby modulating their trafficking to the lymph nodes and their ability to interact with T cells. Furthermore, we show that PLCγ2 is critically required for progression of autoimmune arthritis by affecting DC-mediated T cell priming and focal osteolysis by modulating osteoclast responses.

## Results

### PLCγ2−/− Mice Are Protected from mBSA-Induced Arthritis

**Figure 1:**
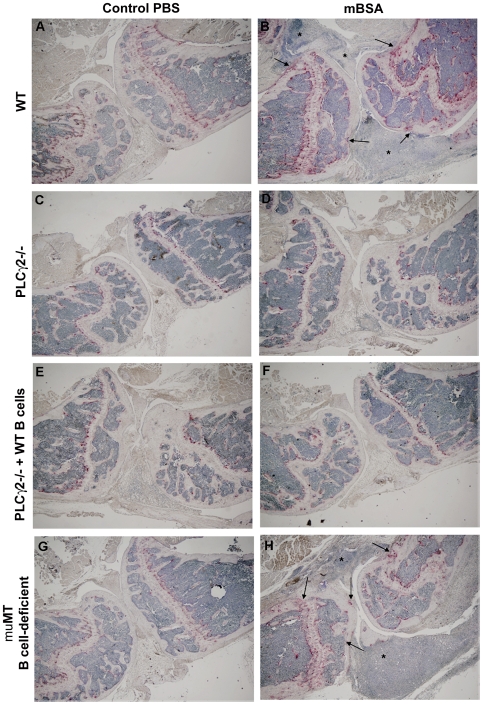
PLCγ2−/− mice are protected from mBSA-induced arthritis independently of B cells. WT (A,B), PLCγ2−/− (C,D), PLCγ2−/− mice transferred with WT B cells (E,F) and muMT B cell-deficient mice (G,H) mice were immunized with mBSA and injected intraarticularly with mBSA to induce arthritis or with PBS as a negative control. Histological sections of control PBS or mBSA injected knees were TRAP stained. Cellular infiltrates (stars), osteoclasts (red cells) and bone erosion (arrows) are depicted. Images were taken using a Nikon inverted microscope and a CoolSnap camera, magnification 20×.

**Figure 2:**
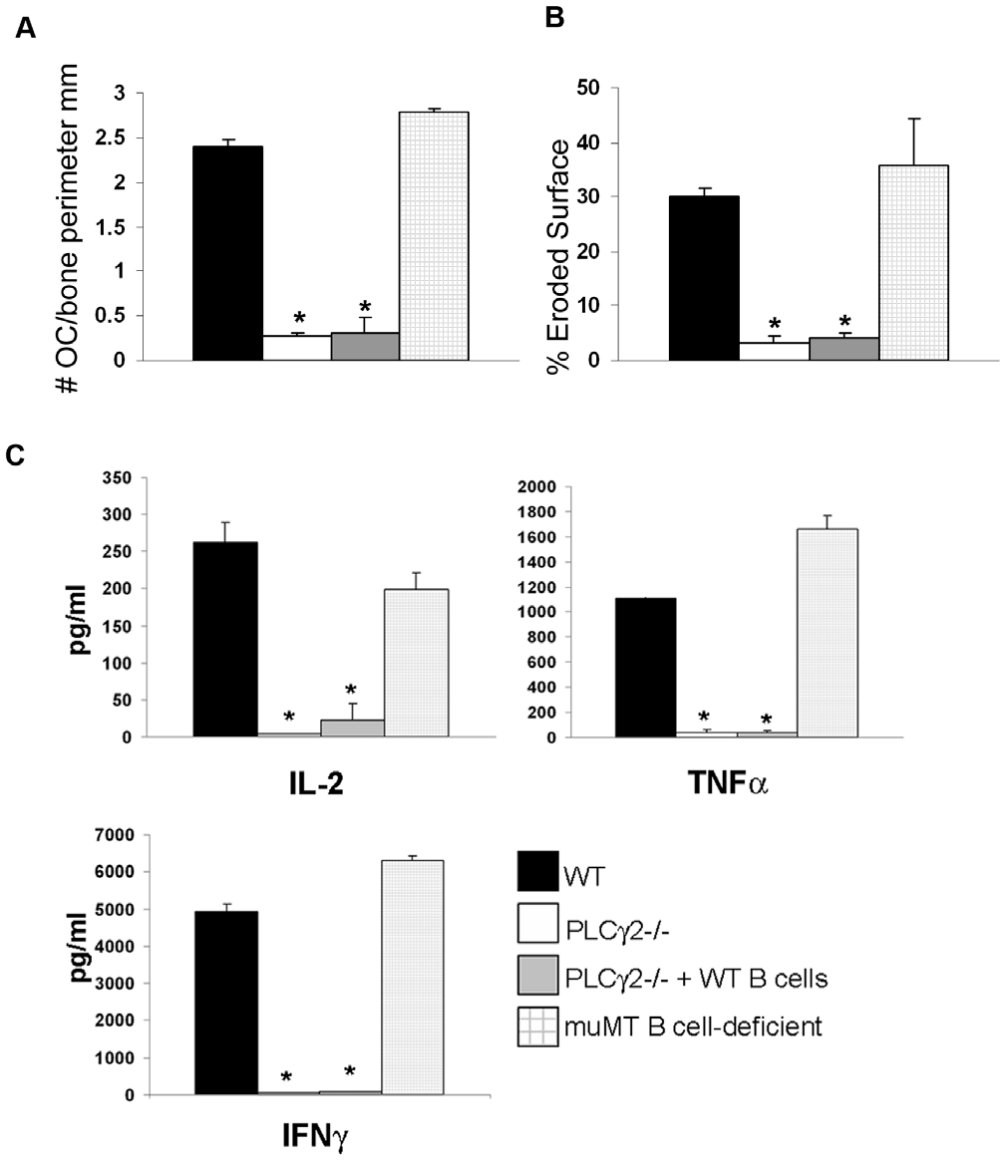
PLCγ2−/− mice display defective osteoclast recruitment and T cell activation in response to mBSA. (A–B) Histomorphometric analysis was performed on knee sections using the metamorph software. Number of osteoclasts for bone perimeter (A) or percentage of eroded surface (B) are depicted. Stars indicate a significant difference of p<0.005. (C) Inguinal lymph nodes were isolated from WT, PLCγ2−/− or muMT B cell deficient mice after induction of arthritis and cultured *ex vivo* in the presence of mBSA (50 µg/ml) for 3 days. T cell activation was assessed as release of IL-2, TNFα and IFNγ in the media following restimulation with the antigen. Stars underline statistical significant differences (p value<0.01) (n = 3).

Considering the importance of PLCγ2 in B cell development, neutrophil function and osteoclastic bone resorption [14], [15], [16], [17], [18], [20], we hypothesized that PLCγ2 could be central to both the inflammatory and the osteolytic response associated with the arthritic disease. In order to define the potential role of PLCγ2 in the development of inflammatory arthritis we employed an established model of antigen-induced arthritis (AIA), in which WT and PLCγ2−/− mice were immunized with methylated BSA (mBSA), followed by induction of acute local inflammatory response by a single knee injection of mBSA (or PBS as a negative control). Induction of arthritis in WT mice led to severe local joint inflammation, characterized by pronounced cellular infiltration and osteoclast recruitment with associated bone destruction compared to PBS control knees (Figure 1A,B and 2A,B). In contrast, PLCγ2−/− animals showed no clinical signs of knee swelling, undetectable cellular infiltration or pannus formation in the joint space, and no osteoclast recruitment or bone erosion following the local injection of mBSA (Figure 1C,D and 2A,B). The protection of PLCγ2−/− mice from induction of arthritis was not due to absence of mature B cells, since PLCγ2−/− mice adoptively transferred with WT B cells (Figure S1) were still resistant to development of the disease (Figure 1E,F and 2A,B). In line with this observation, muMT mice, which lack B cells, developed inflammation in response to mBSA and showed increase OC number and signs of bone erosion as their WT counterpart (Figure 1G,H and 2A,B).

T cells are the primary players during the inductive phase of inflammatory arthritis [21]. To increase our understanding of the impaired inflammatory response observed in PLCγ2−/− mice, we analyzed T cell activation after induction of the disease. Paralleling the massive cellular infiltrate observed in the mBSA injected knees of WT and muMT B cell-deficient mice, T cells isolated from inguinal lymph nodes of these animals released IL-2, TNFα and IFNγ when restimulated in vitro with mBSA (Figure 2C). In contrast, mBSA-dependent production of T cell cytokines in samples from PLCγ2−/− mice or PLCγ2−/− mice adoptively transferred with WT B cells was barely detectable (Figure 2C), suggesting a requirement of PLCγ2 for correct T cell activation in vivo. Importantly, PLCγ2−/− T lymphocytes can release inflammatory cytokines when activated in vitro with PMA and ionomycin (Figure S2), confirming that PLCγ2 does not directly control T cell function [22], [23]. Thus, deletion of PLCγ2 confers protection against the development of mBSA-induced arthritis due to defective antigen-dependent T cell activation.

### PLCγ2 Controls Actin Organization in Mature DCs

**Figure 3:**
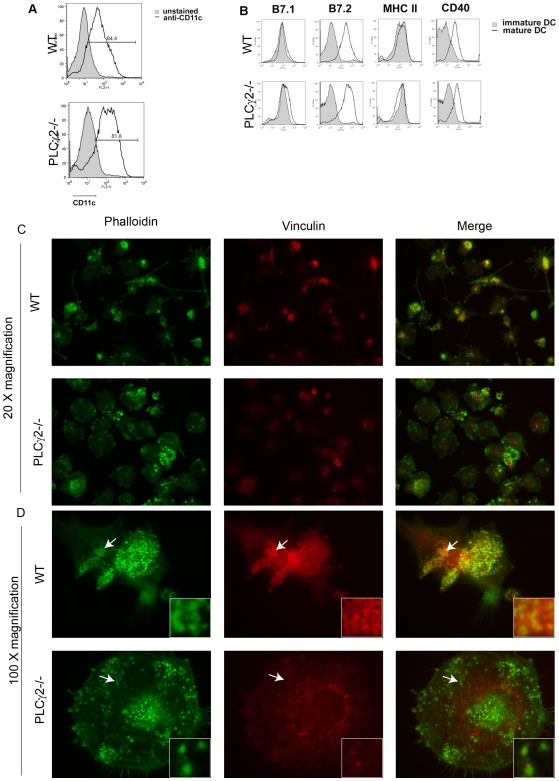
PLCγ2−/− DCs present normal maturation markers but abnormal actin organization. (A–B) Bone marrow derived DCs were stained with CD11c FITC and analyzed for purity by FACS after 9 days in culture with GM-CSF (A). Immature or mature DCs were stained with CD11c FITC or CD11c PE together with H-2^d^ FITC, CD80 FITC, CD86 PE, CD40 PE and analyzed with FACS (B). (C–D) Cytoskeletal organization was visualized in LPS-matured DCs by staining actin (green) and vinculin (red) using 20× (C) or 100× (D) magnification. In WT cells podosomes appear as dotted actin structures surrounded by a ring of vinculin (arrow indicates the podosome structures that are enlarged in the box on the right). In contrast, in PLCγ2−/− DCs, the actin dots are not surrounded by the belt of vinculin (see arrow and enlarged box).

DCs are potent inducers of naïve T cell responses [1]. We hypothesized that PLCγ2 might regulate T cell activation by affecting DC maturation or function. To determine if PLCγ2 was required for development of DCs, we generated WT and PLCγ2−/− DCs from bone marrow precursors in the presence of GM-CSF (Figure 3A). Expression of CD11c and upregulation of maturation markers (CD80, CD86, CD40) in response to LPS stimulation were similar between WT and mutant cells (Figure 3B), as well as expression of MHC I (not shown) and MHC II molecules (Figure 3B). Morphologically, consistent with LPS-induced DC maturation, WT DCs assumed an elongated shape, with numerous filamentous membrane extensions protruding from their cell bodies (Figure 3C top panel). Enrichment of podosomes, short lived dotted-like actin structures (Figure 3D top panel; in green) surrounded by a ring of vinculin (Figure 3D top panel; in red), were also seen at the protruding body of WT cells. In contrast, PLCγ2−/− DCs appeared larger and more round (Figure 3C bottom panel). Actin staining (in green) revealed absence of long membrane extensions, and fewer number of abnormal podosomes (Figure 3D bottom panel; in green), lacking the external ring of vinculin (Figure 3D bottom panel in red) and a specific cellular localization as observed in WT DCs. Thus, while not affecting expression of DC maturation markers, PLCγ2 modulates actin reorganization in mature DCs.

### PLCγ2 Controls DC Migration by Affecting CCR7 Signaling and Rac Activation

**Figure 4:**
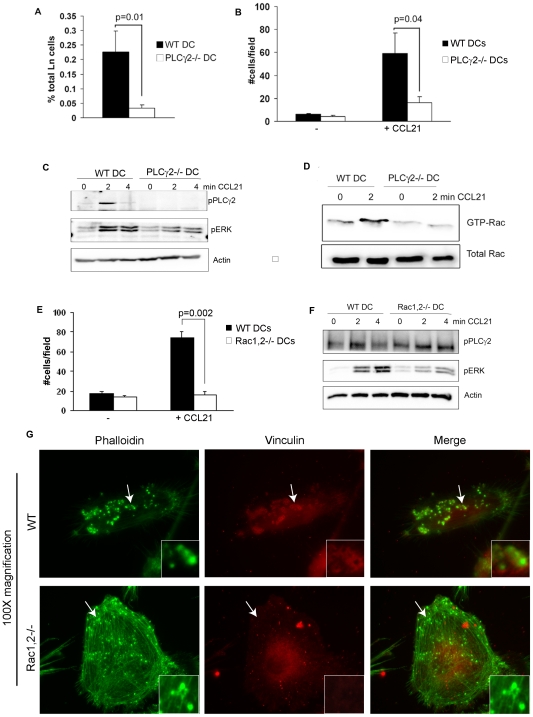
PLCγ2 is required for DC migration by regulating CCL21 signaling. (A) In vivo trafficking of mature WT and PLCγ2−/− DCs to the lymph nodes was assessed in a competitive homing assay. Low CFSE-labeled WT and high CFSE-labeled PLCγ2−/− DCs were coinjected in a 1∶1 ratio into the footpad of WT mice and their recruitment to the draining popliteal lymph nodes was analyzed 2 days later as presence of CFSE+DCs. Data represent the mean number of recovered cells +/− standard deviation (n = 4). (B) In vitro migration of mature WT and PLCγ2−/− DCs was evaluated in response to CCL21 using transwell chambers. (C) WT or PLCγ2−/− mature DCs were stimulated with CCL21 for different time points and activation of PLCγ2 and ERK was evaluated by Western Blot. (D) Western blot analysis of active Rac1 pulldown was performed in WT and PLCγ2−/− DCs stimulated with CCL21 for 2 minutes. (E) In vitro migration of mature WT and Rac1/2−/− DCs was evaluated in response to CCL21 using transwell chambers. (F) WT or Rac1,2−/− mature DCs were stimulated with CCL21 and protein lysates were subjected to Western Blot analysis. Each WB figure is representative of 3 independent experiments. (G) Actin structures were visualized in mature WT or Rac1,2−/− DCs by staining of actin (green) and vinculin (red). Magnification 100×. Podosomes depicted by arrows are enlarged in the box.

Podosomes are dynamic actin structures, typical feature of highly motile cells[9], [24], [25]. The aberrant podosome organization observed in PLCγ2−/− DCs suggested a role for PLCγ2 in cell motility. To evaluate this possibility, we employed a competitive homing assay in which we coinjected a 1∶1 mixture of differentially labeled WT (low CFSE) and PLCγ2−/− (high CFSE) DCs into the footpad of WT mice and analyzed by FACS analysis the number of cells reaching the draining popliteal lymph nodes 2 days later. Surprisingly, we observed that PLCγ2−/− DCs had significantly reduced ability to reach the lymph nodes compared to WT cells (Figure 4A and Figure S3). A similar result was observed when WT DCs were labeled with high CFSE and PLCγ2−/− DCs with low CFSE (not shown). Signs of defective migration were also confirmed in vitro, when we monitored the chemotactic response of DCs towards CCL21 (Figure 4B). While WT DCs efficiently migrated trough the pore of the transwell chamber (59+/−17 cells/field), the number of migrating PLCγ2−/− DCs was about 3 fold decreased (16+/−5 cells/field). Consistent with impaired migration, PLCγ2 was activated downstream of CCR7 G Protein Coupled Receptor, as activation of ERK was abolished in PLCγ2−/− DCs stimulated with CCL21 (Figure 4C). In line with the cytoskeletal abnormalities, activation of the small GTPase Rac was significantly dampened in PLCγ2−/− DCs in response to the chemokine (Figure 4D). Similarly to PLCγ2−/− DCs, Rac1,2−/− DCs fail to migrate in response to CCL21 (Figure 4E) due to impaired CCR7 activation, as ERK phosphorylation in response to CCL21 was significantly dampened in the double KO cells (Figure 4F). However, PLCγ2 was normally phosphorylated in Rac1,2−/− DCs (Figure 4F), indicating that PLCγ2 modulates DC motility via Rac activation downstream of CCR7 receptor.

To further explore the possibility that Rac modulates podosome organization in DCs, WT and Rac1/2−/− DCs matured with LPS were stained for actin and vinculin. The majority of Rac1/2−/− DCs lack podosomes (86+/−2% cells compared to only 25+/−7% cells without podosomes in WT DCs). Similarly to PLCγ2 deficient DCs, the few cells that display dotted-like actin structures which resemble the podosomes observed in WT cells (Figure 4G top panels, arrow points to podosomes), lack the ring of vinculin surrounding the actin core (Figure 4G bottom panels).

### PLCγ2 Is Required for DC∶ T Cell Interaction

**Figure 5:**
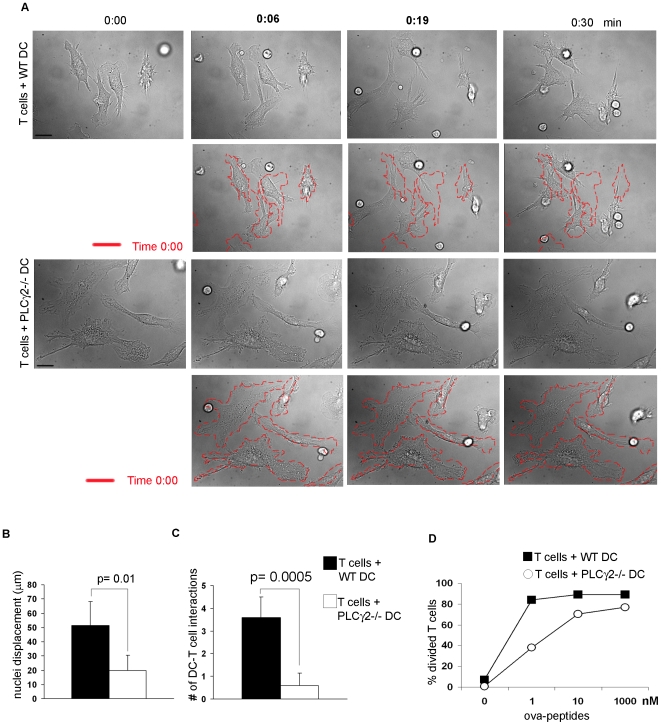
PLCγ2 is required for DC∶T cell contacts and T cell priming in vitro. (A) Antigen-pulsed WT or PLCγ2−/− DCs were cocultured with OT-II T cells and DC∶T cell interactions were filmed for 30 minutes using time lapse microscopy. In panel A, individual frames from Movie 1 are reported, together with superimposition of the initial frame (time 0:00) in red. Scale bar 10 µm. Magnification 60×. (B) WT and PLCγ2−/− DC mobility was quantified by measuring nuclei displacement over time. Position of nuclei was measured every 2.5 minutes for 30 minutes. Figure represents the average space traveled by nuclei of different cells +/− standard deviation. Analysis was conducted using methamorph software. (C) Number of DC∶T cell interactions was counted as the average number of contacts formed by different DCs with T cells +/− standard deviation. (D) CFSE-labeled OT-II T cells were cocultured with WT or PLCγ2−/− DCs in the presence of different concentrations of ovalbumin-peptides. After 3 days T cell proliferation was assessed as CFSE dye dilution. The figure is representative of 3 independent experiments.

Podosomes are active dynamic structures involved in cell migration [9], [24], [25] and cell∶cell interactions [26]. Once DCs reach the lymph nodes, they need to make contacts with potentially reactive T cells to initiate T cell dependent responses. In this phase a single DC can interact and activate multiple reactive T cells. Thus, we wondered if PLCγ2 was also modulating the formation of DC∶T cell contacts occurring during T cell priming, a process known to be mediated by Rac [13]. To test this hypothesis, we used time-lapse video microscopy to monitor the interactions between antigen-loaded DCs with T cells. While WT DCs appeared highly dynamic, projecting numerous membrane extensions in the direction of the T cells (Figure 5A and Movie S1), PLCγ2−/− DCs were poorly motile, well anchored to their initial position, and extended only few dendrites towards T cells (Figure 5A and Movie S2). The quantification of DC cell movement, as determined by nuclei displacement, clearly showed that PLCγ2−/− DCs failed to move towards the surrounding T cells (Figure 5B). Furthermore, lack of directional cell body movements or polarization towards adjacent T cells also affected the number of DC∶T cell interactions which were barely detectable in the presence of PLCγ2−/− DCs (Figure 5C).

We next tested the ability of PLCγ2−/− DCs to prime T cells in vitro, by culturing WT or PLCγ2−/− DCs together with OT II T cells and increasing concentrations of the cognate peptide for ovalbumin. Paralleling the observation that PLCγ2 modulated DC∶T cell interactions, PLCγ2−/− DCs were less efficient than WT DCs in inducing T cell proliferation, as determined by CFSE dye dilution (Figure 5D).

### PLCγ2 Modulates DC-Mediated T Cell Activation *In Vivo*

**Figure 6:**
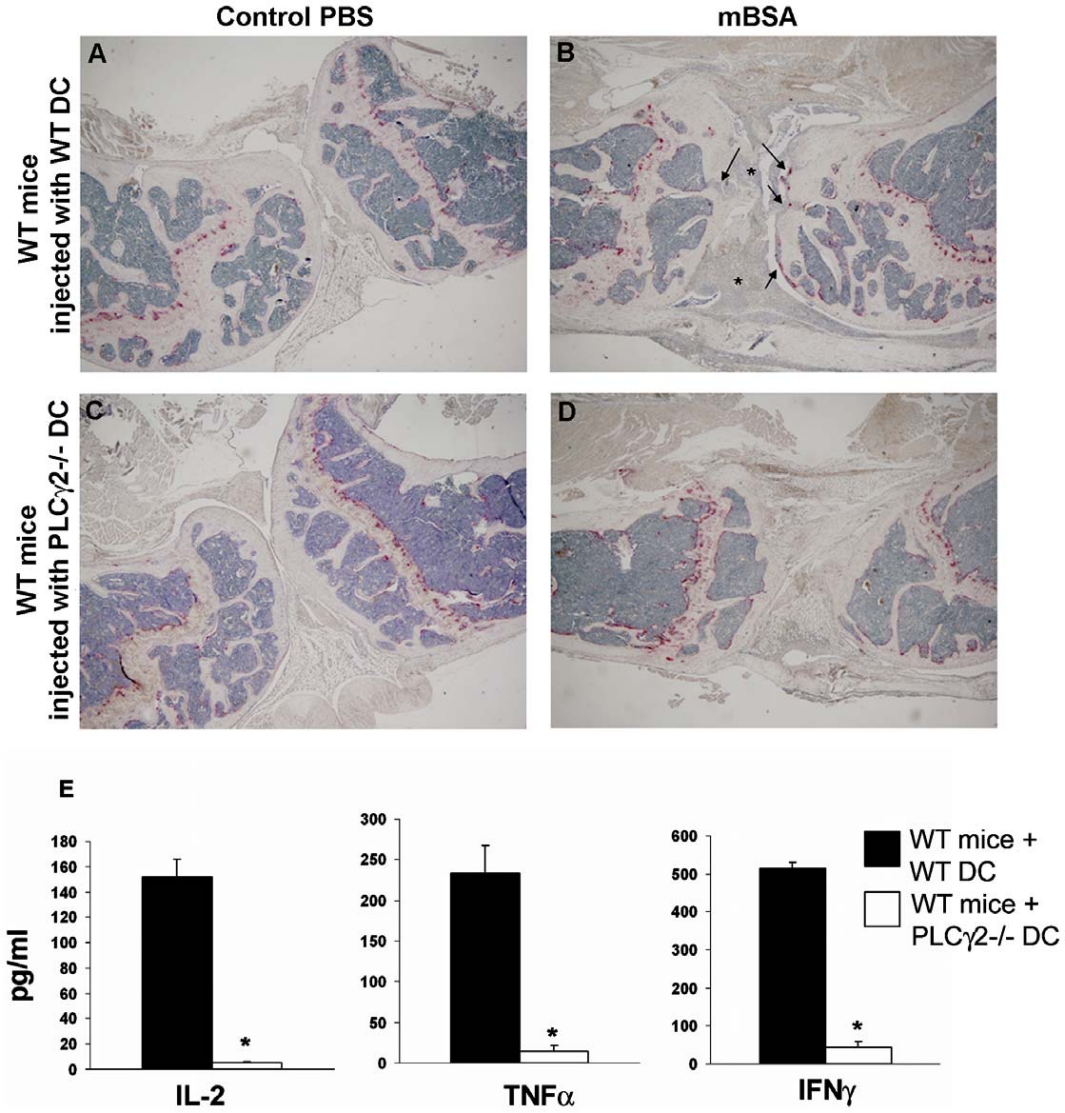
PLCγ2−/− DCs fail to induce arthritis when injected into WT mice. (A–D) WT mice were immunized with mBSA-pulsed WT (A,B) or PLCγ2−/− (C,D) DCs followed by intraarticular injection of mBSA to induce local inflammation or PBS as a negative control. Histological analysis of mBSA-injected knee reveals insurgence of arthritis as indicated by presence of inflammatory infiltrates (stars), osteoclast recruitment (TRAP-stained red cells) and bone erosion (arrows) in WT animals injected with WT but not PLCγ2−/− DCs. Images were taken using a Nikon inverted microscope and a CoolSnap camera, magnification 20×. (E) Inguinal lymph nodes of mice treated as above were cultured *ex vivo* with mBSA (50 µg/ml) for 3 days and T cell activation was determined as release of IL-2, TNFα and IFNγ in the culture medium. Statistical significant differences are underlined by stars (p value<0.01) (n = 3).

**Figure 7:**
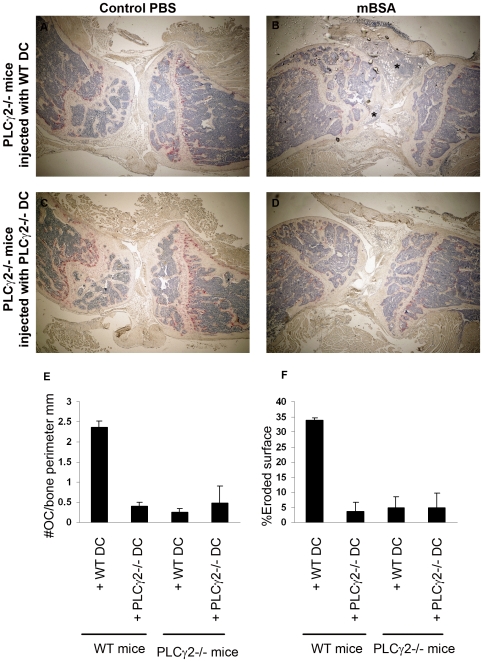
WT DCs rescue inflammation but not focal osteolysis when injected in PLCγ2−/− mice. (A–D) PLCγ2−/− mice were immunized with mBSA-pulsed WT (A,B) or PLCγ2−/− (C,D) DCs followed by intraarticular injection of mBSA to induce local inflammation or PBS as a negative control. Inflammatory infiltrates in B are depicted by stars. Magnification 20×. (E,F) Histomorphometric analysis was performed on knee sections of both WT or PLCγ2−/− mice injected with WT and PLCγ2−/− DCs. Number of osteoclasts for bone perimeter (E) or percentage of eroded surface (F) was calculated using metamorph software.

To explore the in vivo relevance of our findings, we then analyzed the capacity of PLCγ2−/− DCs to trigger a T cell response in a variant of AIA model evoked by administration of antigen-loaded DCs. Specifically, WT recipient mice were immunized with mature mBSA-pulsed WT or PLCγ2−/− DCs on days 0 and 7, followed by a local knee injection of mBSA on day 21. In this context, only exogenously injected DCs have the ability to present the antigen to endogenous WT T cells. When WT mice were immunized with mBSA-pulsed WT DCs, they developed visible signs of arthritis. Cellular infiltrates, pannus formation and local bone erosion were visible in the mBSA injected knee, and such changes were not detected in the control knee injected with PBS (Figure 6A,B and 7E,F). In contrast, development of the disease did not occur in WT mice injected with mBSA-pulsed PLCγ2−/− DCs (Figure 6C,D and 7E,F). Consistent with the histological findings, T cells isolated from mice immunized with PLCγ2−/− DCs failed to release T_H_ cytokines when restimulated *ex-vivo* with mBSA (Figure 6E). Importantly, injection of WT DCs into PLCγ2−/− mice was sufficient to rescue the inflammatory response, leading to massive cellular infiltrate in the joint space (Figure 7). Confirming the previously known role of PLCγ2 as critical mediator of osteoclastic formation and function, PLCγ2−/− mice injected with WT DCs were still protected from osteoclast recruitment in the knee space and focal osteolysis (Figure 7). Collectively, these data suggest a critical role for PLCγ2 in inflammatory arthritis by affecting both DC-mediated T cell activity and osteoclastic responses.

## Discussion

In the past decade, the focus of rheumatoid arthritis (RA) research has been to determine which cytokines and inflammatory mediators are produced at the site of disease. However, biological pharmacotherapy aimed at blocking the activity of inflammatory cytokines is not completely effective in controlling symptoms and disease progression [27]. We are now beginning to understand that migration of inflammatory cells into the joints and DC∶T cell contacts are other important components of the disease, specifically because adhesion molecules not only modulate tissue infiltration, but also affect cell activation and cell-cell interactions. Here we have identified PLCγ2 as a critical component of the immune response associated with RA by affecting DC trafficking from peripheral tissues to the lymph nodes and DC∶T cell interactions required to initiate the local inflammation via modulation of actin dynamics.

Despite the well known role of PLCγ2 in B cell maturation [17], [18], PLCγ2 is also required for osteoclast differentiation and function [15], [16]. Indeed, PLCγ2 deletion leads to an osteopetrotic phenotype in vivo [16]. More recently, we have also found that PLCγ2−/− mice are protected from inflammation in the serum transfer arthritis model [14], which is primarily dependent on neutrophil recruitment and activation [28]. However, human rheumatic disease is more complex, involving multicellular responses and not only neutrophil infiltration in the joint space. We now demonstrate that PLCγ2−/− mice are also protected from development of inflammation and tissue damage in a T cell dependent model of arthritis, which more closely resembles the complex pathophysiology of the human disease. Using mBSA as antigen to induce arthritis, we found impaired T cell activation in PLCγ2−/− mice. However, T cells do not express PLCγ2, excluding the possibility that PLCγ2 directly affects T cell function [22], [23]. B cells, which fail to fully mature in the absence of PLCγ2 [17], [18], are neither the source of the defective T cell response, since WT B cell transplant into PLCγ2−/− mice failed to rescue the inflammatory response to mBSA. Additionally, muMT B cell-deficient mice could develop the arthritic disease to the same extent as WT animals. Thus, other inflammatory cells modulating T cell activation are affected by the deletion of PLCγ2.

In the complex puzzle of arthritis, DCs are known to play a crucial role as they are the most potent antigen presenting cells in T cell responses [29], [30]. When PLCγ2−/− DCs were loaded with the antigen *ex vivo* and injected into WT animals, they were unable to induce inflammation or support T cell activation, although similarly treated WT DCs were fully competent in the same assays. In contrast, injection of WT DCs into PLCγ2−/− mice fully restored the arthritic inflammatory response. Two distinct events modulate the capacity of DCs to regulate T cell activation: first, their ability to migrate from peripheral tissues to secondary lymphoid organs upon antigen binding, and second, the interaction with and activation of T cells in lymph nodes [31], [32], [33]. We found that PLCγ2 controls both events.

Migration of DCs to the lymph nodes is critical for induction of T cell responses [4]. Lymph node homing of plasmacytoid DCs has been suggested to be critical for regulatory T-cell development and tolerance induction in a cardiac allograft transplant model [34]. Our data clearly show that in absence of PLCγ2 DCs fail to home to the lymph nodes and PLCγ2−/− mice do not develop inflammatory arthritis. Even in the circumstance that some PLCγ2−/− DCs could reach the secondary lymphoid organs, the in vitro imaging data suggest that lack of PLCγ2 affects DC ability to extend ruffles and long dendrites towards the adjacent T cells. Consequently, PLCγ2−/− DCs display defective T cell priming in vitro and ex-vivo. We cannot rule out the possibility that PLCγ2 may also play a role in additional DC functions occurring during T cell priming, such as presentation of the antigen or production of cytokines [19], [35], [36] that support Th cell differentiation. However, trafficking to the lymph nodes is an event that occurs in the initial phase of T cell priming and DC∶T cell contacts precede T cell activation.

Mechanistically, these two, albeit distinct, DC functions require active cytoskeleton rearrangements, as membrane protrusions must form to activate the migratory machinery as well as to favor cell∶cell contacts. Although cell maturation occurs normally in the absence of PLCγ2, the null cells appear larger, lack long membrane elongations that DCs form to interact with T cells, and present abnormal podosome structures, characterized by condensed actin dots not surrounded by the typical ring of vinculin. We have previously reported a role for PLCγ2 in actin dynamics in osteoclasts. PLCγ2−/− osteoclasts, like DCs, have abnormal actin cytoskeleton, as they lack the typical belt of actin which enables the cell to seal the acidic environment of the resorptive lacunae, allowing bone dissolution. As a consequence of aberrant resorption, PLCγ2−/− mice have increased bone mass in basal condition and now we show are also protected from inflammatory-induced bone loss. Similarly to the osteoclasts, we find that DCs have an abnormal podosome structure. It is not clear how PLCγ2 specifically modulates formation of podosomes, as focal contacts, more stable adhesive structures, form normally in PLCγ2−/− DCs following short exposure to LPS stimulation (Figure S4). In the osteoclasts, PLCγ2 regulates c-Src activation and membrane localization and c-Src null osteoclasts lack podosomes[15]. It is possible that a similar mechanism occurs in DCs, as vinculin, a Src substrate [37], fails to surround the podosome actin core. Lack of these highly dynamic structures can affect the capacity of the cell to rapidly rearrange the actin cytoskeleton during cell migration or cell∶cell contacts. Evidence showing the importance of podosome organization in DC function comes from Wasp deficient DCs, which lack podosomes, and display trafficking defects [38] and impaired T cell activation [39]. Similarly, WASP-interacting-protein (WIP) deficient DCs lack the typical belt of vinculin around the actin core of the podosome, fail to polarize and form stabilized leading edges in response to chemokine gradient and consequently are poorly motile [25]. However the physiological importance of these actin-regulating molecules in DC function in a disease context in vivo has never been reported.

Activation of the small Rho GTPase Rac is also essential for cell polarization and membrane protrusions at the leading edge of migratory cells [10], [11], [12], [13]. We now provide new evidences that Rac deficiency impairs podosome formation in LPS matured DCs. Importantly, Rac1,2−/− DCs have severe migratory defects and cannot extend polarized dendrites towards T cells [13], thereby fail to promote T cell activation, similarly to what we observed in PLCγ2−/− DCs. Mechanisms of Rac activation in DCs are unclear. In plasmacytoid, but not myeloid DCs, DOCK2 controls cell migration by functioning downstream of chemokine receptors and activating Rac [40]. Direct interaction between constitutively active Rac and the split pleckstrin domain of PLCγ2 has been recently described in COS cell line [41]. Indeed, we find Rac to be activated in response to the chemokine CCL21 and regulated by PLCγ2 in bone marrow derived DCs. Conversely, PLCγ2 phosphorylation is normal in Rac1/2 deficient DCs. Thus, our data position PLCγ2 as a critical modulator of Rac activity downstream of CCL21 receptor, CCR7. This data are further supported by the similar morphological, functional and signaling defects observed in both PLCγ2−/− and Rac1/2−/− DCs.

DCs constitute a unique link between innate and adaptive immunity, and their targeting during inflammatory conditions may represent a valid alternative to current therapies which are often specific for only one component of the immune response [27]. Our in vivo animal studies unequivocally support the importance of PLCγ2 in DCs during the development of an inflammatory response associated with antigen-induced arthritis. Moreover, previous studies showed that activation of PLCγ2 in DCs occurs downstream of several immune receptors, in particular TLR4 [19] and Dectin-1[36], [42]. Altogether, these data suggest that PLCγ2 is actively regulated in DCs during the course of an inflammatory response.

We have previously reported that neutrophil activation is impaired in PLCγ2−/− mice [14]. Although we cannot exclude that neutrophils also participate in the development of inflammation in the antigen-induced model of arthritis, the finding that injection of antigen-pulsed PLCγ2−/− DCs in WT mice is not sufficient to initiate the inflammatory disease, highlights the importance of DC motility and actin dynamics during DC-mediated activation of T cell responses. In agreement with this posture, injection of WT DCs in PLCγ2−/− mice rescues inflammation, however bone resorption is still impaired. Focal osteolysis is a deleterious consequence of arthritic inflammatory conditions, reflected as bone pain, periarticular and appendicular osteopenia, and impaired mobility. Here we provide evidences that PLCγ2 modulates bone loss independent from inflammation. While our prior investigation has revealed a role for PLCγ2 in osteoclast formation and function under basal condition, now we prove that this holds true even in the context of an inflammatory condition known to be favorable for osteoclastic recruitment.

In summary, the importance of PLCγ2 in osteoclasts [15], [16] and neutrophils [14], [20], in conjunction with its role in DC-mediated T cell activation here discussed, positions the molecule as a point of convergence between bone and immune systems in complex pathologies such as RA and other autoimmune diseases characterized by an interplay between bone and immune cells.

## Methods

### Mice

PLCγ2−/− mice have been previously described [18]. CD45.1 B6 congenic mice were purchased from Jackson lab. muMT B cell-deficient mice and OT-II mice were kindly provided from Dr. Virgin at Washington University. Rac1,2−/− cells were isolated from dKO conditional mice, kindly provided by Dr. Ross at Washington University (Rac2−/− mice X Rac1 ^fl/fl^/LysoM cre+/+ mice). All experiments were approved by the Washington University School of Medicine animal care and use committee. All the mice used in the experiment came from a B6 background.

### mBSA-Induction of Arthritis

Mice were immunized on day 0 and day 7 by intradermal injection at the base of the tail of 100 µg mBSA (Sigma-Aldrich) emulsified in 0.1 ml Freund's adjuvant (CFA on day 0 and IFA on day 7, BD). At the same time 1 µg pertussis toxin (List Biological Laboratories Inc.) was also injected intraperitoneally. Arthritis was induced on day 21 by intraarticular injection of 100 µg of mBSA in 10 µl PBS into the right knee, while the contralateral knee was injected with PBS alone as a negative control. Mice were sacrificed on day 32 and insurgence of arthritis was assessed by histological examination of knee sections by H&E and TRAP staining.

### B Cell Transfer

Splenic cells from WT CD45.1 congenic mice were depleted of erythrocytes in red cell removal buffer (154 mM NH_4_Cl, 0.1 mM EDTA, 10 mM NaHCO_3_) and B cells were purified using CD19 immunobeads (Mylteny Biotech). 20*10^6^ purified B cells were injected into the tail vein of PLCγ2−/− mice 1 day before the mBSA immunization protocol was started. Presence of B cells in the blood was assessed at day 21 by FACS analysis for B220+CD45.1+ cells.

### BMDC Isolation

DCs were isolated from total bone marrow cells harvested from long bones and cultured for 7–10 days in complete medium supplemented with 2% GM-CSF, as titrated from supernatants of the GM-CSF secreting TOPO cell line. DCs were matured by overnight treatment with 1 µg/ml LPS (Sigma-Aldrich) and, where appropriate, pulsed with mBSA [50 µg/ml] for 4 hours prior to maturation. For FACS analysis of DC cultures, the following antibodies were utilized: CD11c FITC, CD11c PE, CD80 FITC, CD86 PE and CD40 PE (from BD), H-2^d^ FITC (from eBioscience).

### Induction of Arthritis by DC Transfer

WT or PLCγ2−/− DCs were pulsed with mBSA for 4 hours and then matured o.n. with LPS. Recipient mice were then immunized at day 0 and 7 by s.c. administration of these mBSA-pulsed DCs (2*10^6^ cells into each footpad). On day 21, arthritis was induced by injection of mBSA [10 µg/µl] into the right knee, while controlateral knee received PBS. Mice were sacrificed on day 32 and histological examination was performed.

### Restimulation of Draining Lymph Node Cells Ex Vivo

At the end of the in vivo experiments, mice were sacrificed and draining inguinal lymph nodes removed. Single-cell suspensions were prepared, and from each mouse all cells from each lymph node were plated in 1 well of a 96 well/plate in 200 µl of RPMI 1640 containing 10% mouse serum for a total of at least 8 wells. Cells were restimulated with 50 µg/ml mBSA in vitro for 3 days. Supernatants were harvested after 3 days and cytokine production was assessed using the T_H_1/T_H_2 assay kit from BD bioscience according to the manufacture. Briefly, three bead populations with distinct fluorescence intensities and coated with capture antibodies specific for IL-2, IFN-γ or TNF-α were mixed with the PE-conjugated detection antibody and then incubated with samples or standards for 2 hours. After washing, the samples were analyzed with a FACScalibur and mean fluorescence was evaluated.

### Immunofluorescence

DCs were seeded on glass coverslips at a concentration of 2*10^5^ cell/ml and matured o.n. with LPS treatment. After maturation, cells where fixed in 4% PFA (Polyscisnces), stained with a mouse anti-vinculin antibody (Sigma) followed by incubation with a secondary anti-mouse 564 antibody together with phalloidin 488 (both from Molecular Probes). Coverslips were analyzed using an Olympus IX70 inverted microscope with either 20X APO, or 100X APO. Images were captured with a CoolSnap camera (Roper Scientific).

### Competitive *In Vivo* DC Homing Assay

DCs from WT or PLCγ2−/− mice were fluorescently labeled with high (2 µM) or low (400 nM) CFSE and coinjected in the ratio of 1∶1 into each footpad of recipient WT mice (2*10^6^ total DCs for each footpad). After 2 days recipient mice were sacrificed, draining popliteal lymph nodes were recovered and analyzed for presence of CFSE+DCs by FACS.

### DC Migration Assay *In Vivo*

5*10^5^ matured DCs were seeded into the upper filter of 24 well transwell plates (8 µm filters, Corning Incorporated) and allowed to migrate towards CCL21 [10 µg/ml] (eBioscience) or control medium. After 2 hours, 1 µg/well PMA was added to the lower compartment for 10 minutes to allow migrated cells to adhere to the bottom of the well. Cells were fixed, stained with crystal violet and counted.

### DC Stimulation, Western Blot and Rac Activation Assay

5*10^6^ mature DCs starved overnight in 2% FBS were stimulated with 10 µg/ml CCL21 (eBioscience). Cells were lysed in RIPA buffer and equal amount of proteins were resolved by SDS-PAGE to detect pPLCγ2 or pERK (all from Cell Signaling). For analysis of activation of Rac, 500 µg of lysate was subjected to active Rac pulldown according to the manufacture protocol (Rac1 activation Kit, Pierce).

### Time-Lapse Video Microscopy

DCs pulsed with ovalbumin-peptides (1000 nM) were seeded on Glass bottom culture dishes (MatTek) at a final concentration of 2*10^5^ cells/ml in L15 medium +10% FBS. After DCs adhered, 2*10^5^ OT-II CD4+T cells were added to the culture. Pictures were taken every 30 seconds using an Olympus IX70 inverted microscope with a 60x APO lens and a CoolSnap camera (Roper Scientific) starting 10 minutes after addition of T cells, for a maximum of 30 minutes. Analysis of DC∶T cell interactions was performed using methamorph software (Universal Imaging Corporation).

### Antigen Presentation Assay

2*10^4^ WT or PLCγ2−/− immature DCs were plated in 96 well/plates and ovalbumin-peptides (from 0 to 1000 nM) were added to the media. CD4+T cells purified from OT-II mice (CD4 immunobeads Mylteni Biotech) were labeled with CFSE (2 µM) and added to the plates at a final concentration of 1*10^5^ cells/well. After 3 days, T cells were recovered, stained with an APC-conjugated CD4 antibody (BD Bioscience) and T cell proliferation was assessed by FACS analysis.

### Statistics

Two-tailed Student's *t* test was used for all comparisons, with a *P* value of <0.05 set as statistically significant.

## Supporting Information

Figure S1 Efficiency of WT B cell transfer in PLCγ2−/− mice. The number of circulating B cells in PLCγ2−/− mice was evaluated 21 days after WT B cell transfer by FACS analysis of peripheral blood. Donor cells were visualized by CD45.1 PE and B220 FITC co-staining. PLCγ2−/− mice not injected served as a negative control. One representative FACS plot analysis is shown.
(0.14 MB TIF)

Figure S2 PLCγ2−/− T cells normally respond to PMA and ionomycin in vitro. CD4+T cells from WT or PLCγ2−/− mice isolated from inguinal lymph nodes were stimulated in vitro with PMA and ionomycin. After 3 days supernatant was recovered and production of T cell specific cytokines determined.
(0.07 MB TIF)

Figure S3 PLCγ2 is required for DC homing to the lymph nodes. In vivo migration of WT and PLCγ2−/− DCs was assessed in a competitive homing assay. Low CFSE-labeled WT and high CFSE-labeled PLCγ2−/− DCs were coinjected (1∶1) into the footpad of WT mice and their recruitment to the draining popliteal lymph nodes was analyzed 2 days later. One representative FACS plot analysis is shown in the figure.
(0.15 MB TIF)

Figure S4 PLCγ2−/− DCs form normal focal adhesion structures in response to short LPS stimulation. Cytoskeletal organization was visualized in WT and PLCγ2−/− DCs after 2 hour stimulation with LPS by staining actin (green) and vinculin (red). Magnification 100×. Focal adhesions are depicted by arrows.
(2.24 MB TIF)

Movie S1 Interactions between WT DCs and T cells. Antigen-pulsed WT DCs were cocultured with OT-II T cells and DC∶T cell interactions were filmed for 30 minutes using time lapse microscopy.
(10.31 MB ZIP)

Movie S2 Interactions between PLCγ2−/− DCs and T cells. Antigen-pulsed PLCγ2−/− DCs were cocultured with OT-II T cells and DC∶T cell interactions were filmed for 30 minutes using time lapse microscopy.
(10.25 MB ZIP)
