# Supplementary figures and images for: Phospholipase C Gamma 2 Is Critical for Development of a Murine Model of Inflammatory Arthritis by Affecting Actin Dynamics in Dendritic Cells
Source: PLoS One. 2010 Jan 27;5(1):e8909. doi: 10.1371/journal.pone.0008909 (PMC2811739; doi:10.1371/journal.pone.0008909)

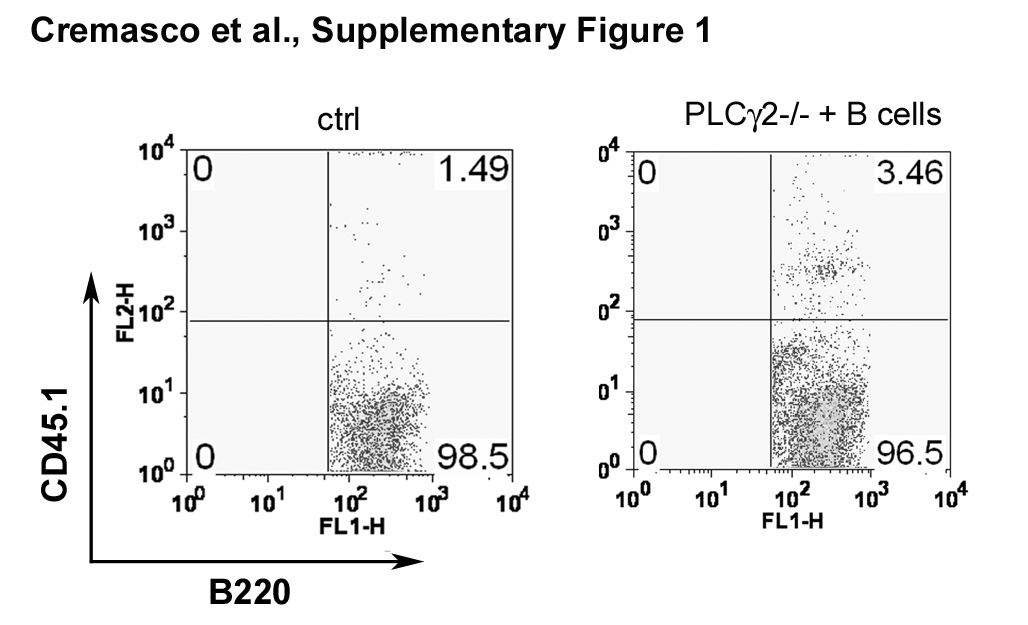

Supplement: Figure S1 — Efficiency of WT B cell transfer in PLCγ2−/− mice. The number of circulating B cells in PLCγ2−/− mice was evaluated 21 days after WT B cell transfer by FACS analysis of peripheral blood. Donor cells were visualized by CD45.1 PE and B220 FITC co-staining. PLCγ2−/− mice not injected served as a negative control. One representative FACS plot analysis is shown. (0.14 MB TIF) [file pone.0008909.s001.tif]

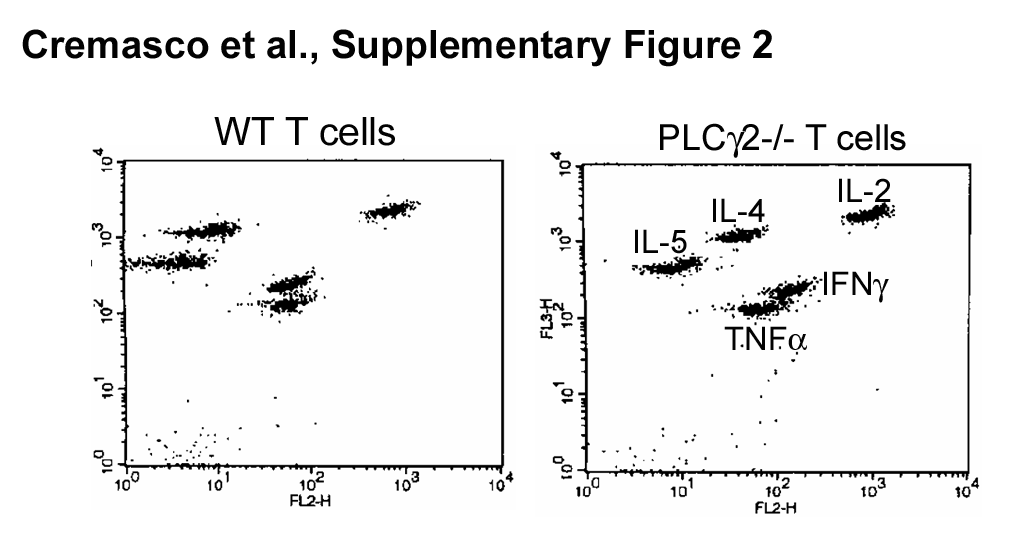

Supplement: Figure S2 — PLCγ2−/− T cells normally respond to PMA and ionomycin in vitro. CD4+T cells from WT or PLCγ2−/− mice isolated from inguinal lymph nodes were stimulated in vitro with PMA and ionomycin. After 3 days supernatant was recovered and production of T cell specific cytokines determined. (0.07 MB TIF) [file pone.0008909.s002.tif]

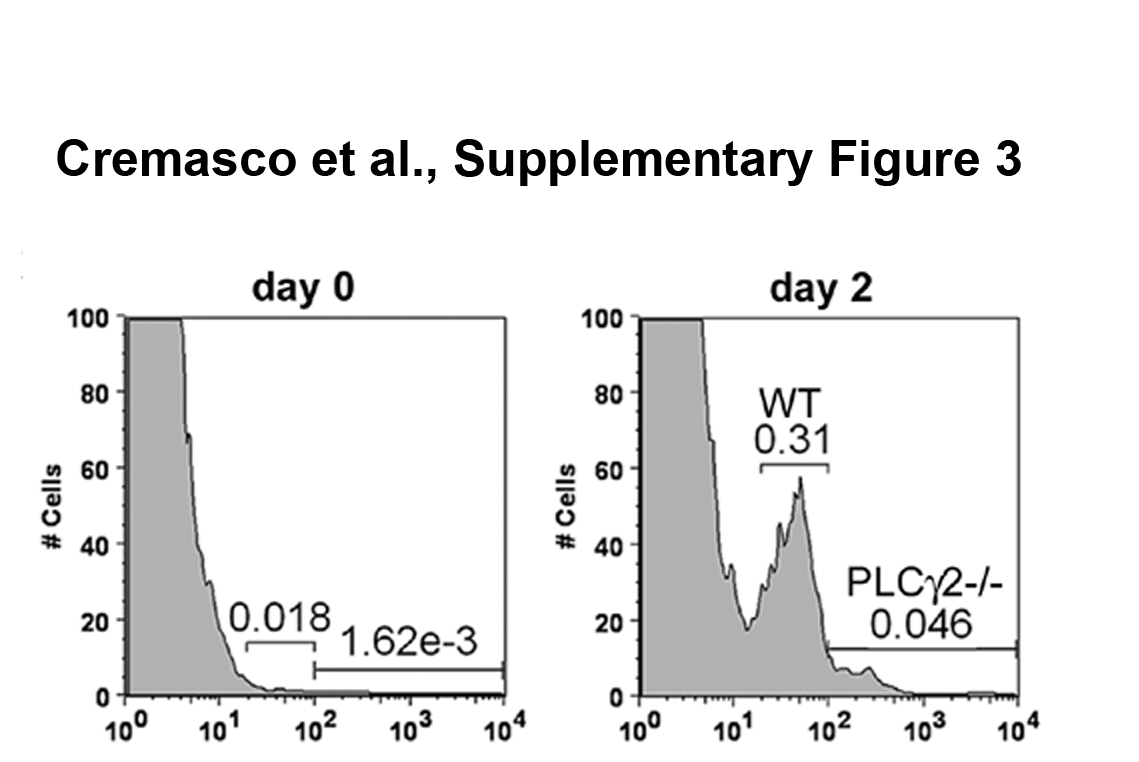

Supplement: Figure S3 — PLCγ2 is required for DC homing to the lymph nodes. In vivo migration of WT and PLCγ2−/− DCs was assessed in a competitive homing assay. Low CFSE-labeled WT and high CFSE-labeled PLCγ2−/− DCs were coinjected (1∶1) into the footpad of WT mice and their recruitment to the draining popliteal lymph nodes was analyzed 2 days later. One representative FACS plot analysis is shown in the figure. (0.15 MB TIF) [file pone.0008909.s003.tif]

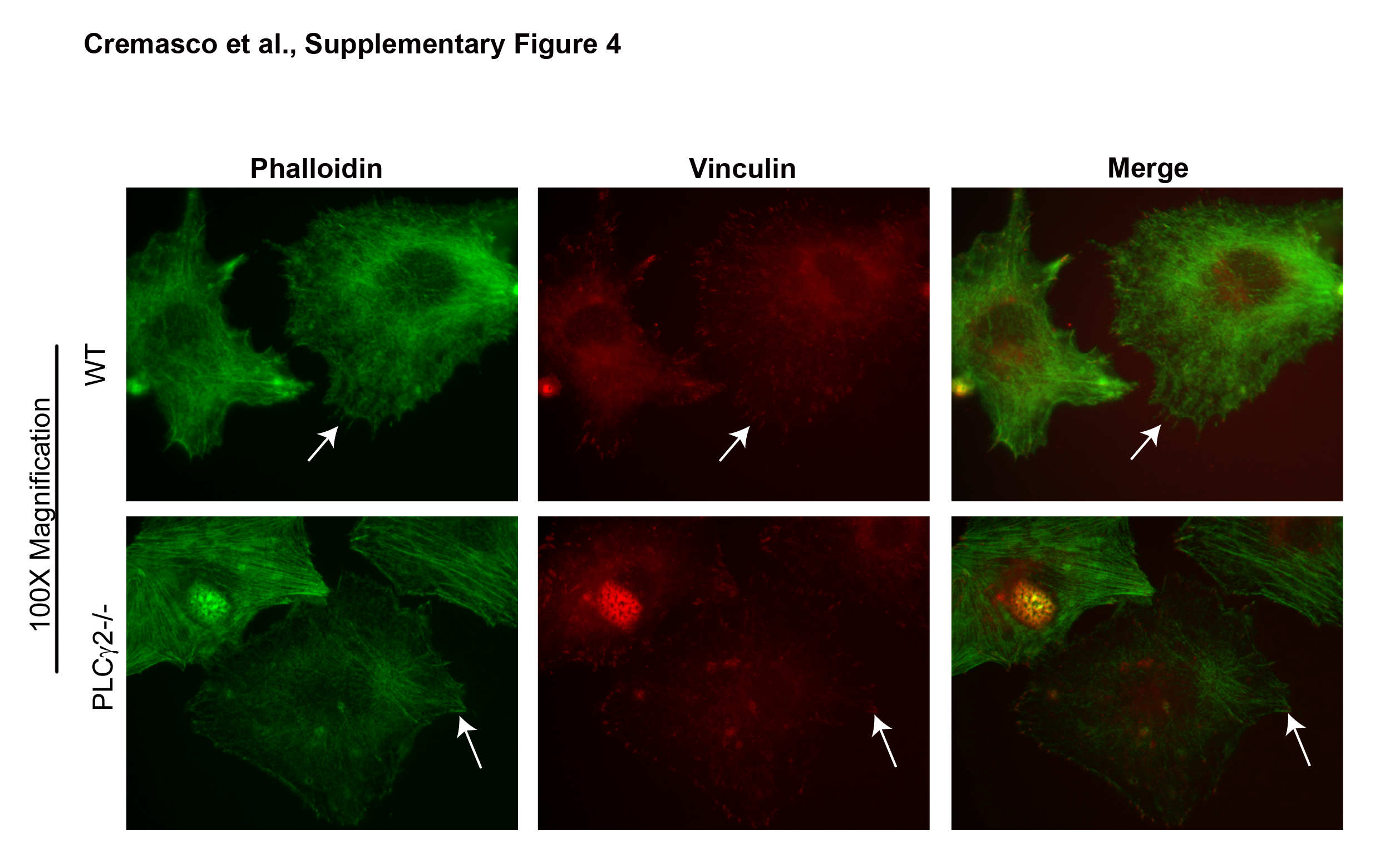

Supplement: Figure S4 — PLCγ2−/− DCs form normal focal adhesion structures in response to short LPS stimulation. Cytoskeletal organization was visualized in WT and PLCγ2−/− DCs after 2 hour stimulation with LPS by staining actin (green) and vinculin (red). Magnification 100×. Focal adhesions are depicted by arrows. (2.24 MB TIF) [file pone.0008909.s004.tif]
